# Co-Occurring Risk Factors for Alcohol Dependence and Habitual Smoking: Results From the Collaborative Study on the Genetics of Alcoholism

**Published:** 2000

**Authors:** Laura Jean Bierut, Marc A. Schuckit, Victor Hesselbrock, Theodore Reich

**Affiliations:** Laura Jean Bierut, M.D., is an assistant professor in the Department of Psychiatry, Washington University School of Medicine, St. Louis, Missouri.Marc A. Schuckit, M.D., is a professor of psychiatry at the University of California, San Diego, School of Medicine, and director of the Alcohol Research Center, San Diego Veterans Affairs Medical Center, San Diego, California.Victor Hesselbrock, Ph.D., is a professor in the Department of Psychiatry, University of Connecticut School of Medicine, Farmington, Connecticut.Theodore Reich, M.D., is a professor in the Department of Psychiatry, Washington University School of Medicine, St. Louis, Missouri

**Keywords:** AOD (alcohol or other drug) dependence potential, smoking, gene expression, dopaminergic receptors, genetic linkage, AODR (AOD related) genetic markers, genetic screening method, chromosome, DNA replication, genetic variance, risk factors

## Abstract

Smoking and alcohol dependence frequently occur together, and both behaviors are determined in part by genetic influences. The Collaborative Study on the Genetics of Alcoholism (COGA), which is investigating the genetic factors contributing to alcohol dependence, also allows for analyses of the genetic factors determining smoking. Using a sample comprised of alcoholics and their closest (i.e., first-degree) relatives as well as a community-based control sample, COGA investigators found that both alcohol dependence and habitual smoking were transmitted within families. This familial transmission resulted from both common and drug-specific influences, which likely include genetic factors. Further genetic studies (i.e., candidate gene studies and genomic screening approaches) have identified several DNA regions that may contain genes that confer a susceptibility for alcoholism. Some of those genes also may contribute to the risk for habitual smoking.

In the United States, excessive alcohol use is a serious public health problem. At some time in their lives, 19 percent of men and 8 percent of women have been diagnosed with alcohol dependence as defined in the American Psychiatric Association’s (APA’s) (1994) *Diagnostic and Statistical Manual of Mental Disorders, Fourth Edition* (DSM–IV). Moreover, 6 percent of men and 3 percent of women have experienced symptoms of alcohol dependence in the past year (Grant and Harford 1995). Unfortunately, the prevalence of alcohol dependence has been increasing among younger people (Chatterji et al. 1997), indicating that alcohol dependence may become an ever more prominent public health problem. In addition to causing numerous serious medical disorders (e.g., liver and heart disease), alcohol dependence is associated with costly, adverse social consequences, such as disruption of families, crime, traumatic accidents, and lost productivity. As a result, the annual costs related to alcohol dependence in the United States for 1998 have been estimated at $185 billion (National Institute on Alcohol Abuse and Alcoholism 2000).

At the same time, 25 percent of the U.S. adult population (i.e., approximately 48 million people) can be classified as current smokers (*a*Centers for Disease Control and Prevention [CDC] 1999*a*). Although the prevalence of smoking has decreased during the past 30 years, little change in smoking rates has occurred since the mid-1990s. More worrisome, smoking rates have increased among adolescents and young adults in the United States, accompanied by a dramatic increase in tobacco use worldwide (CDC 1999*a*). Smoking is a leading cause of preventable disability and death and is associated with multiple well-documented adverse health effects, including heart disease, pulmonary disease, and various cancers. The economic burden of smoking is high. Thus, the annual costs attributable to smoking in the United States have been estimated to be $50 billion in direct medical costs plus a similar amount in lost productivity (CDC 1999*b*).

Alcohol dependence and smoking frequently occur together. Smokers, including those who are nicotine dependent, have an elevated risk for alcohol dependence.^1^ Thus, smokers in general have a 2.1 times greater risk and nicotine-dependent smokers have a 2.7 times greater risk of becoming alcohol dependent compared with nonsmokers (Breslau 1995). Similarly, alcohol-dependent people have a greatly increased risk of smoking (i.e., 4.7 times greater) than do non-alcohol-dependent people (Glassman et al., 1990).

Both alcohol dependence and smoking are complex behaviors that are influenced by environmental as well as genetic factors. Various types of research, including studies of identical and fraternal twins, adoptees, and families with multiple alcoholic members have provided evidence for a strong genetic component in the development of alcohol dependence. Based on these findings, the heritability of alcohol dependence has been estimated at 64 percent (Heath et al. 1997). Similarly, twin studies have supported the role of genetic influences on smoking, suggesting a heritability of 60 percent for nicotine dependence (True et al. 1997). Because alcohol dependence and smoking frequently co-occur and because genetic factors influence both behaviors, twin studies have examined the extent to which both addictions might share some genetic risk factors. These analyses found evidence of a substantial overlap in genetic factors influencing nicotine and alcohol dependence (i.e., a genetic correlation of 0.68)^2^ (True et al. 1999). These data support a common underlying genetic vulnerability to both alcohol and nicotine dependence.

Another study that has assessed the genetic factors contributing to alcohol dependence and has enabled researchers to investigate the relationship of those factors with the genetic factors determining smoking is the Collaborative Study on the Genetics of Alcoholism (COGA). This article provides a brief overview of COGA and its design as well as highlights of some of the study’s results regarding the prevalence of smoking in families of alcohol-dependent people. In addition, the article discusses specific genetic associations between alcohol and nicotine dependence that have been identified by analyzing specific genes (i.e., candidate genes) or by screening study participants’ entire genetic material (i.e., the genome).

## Description of COGA

COGA is a comprehensive study conducted at multiple sites across the United States to detect and map genes that confer a susceptibility for alcohol dependence and related disorders, including dependence on other drugs (e.g., nicotine). The study initially recruited alcohol-dependent people and their family members, thereby obtaining a sample of people at high risk for both alcohol-use disorders (i.e., alcohol abuse and alcohol dependence) and smoking. This sample allows COGA investigators to study the relationship between alcohol dependence and smoking among family members.

To obtain their sample, the investigators first recruited alcohol-dependent patients (also called index cases or probands) who were treated in both inpatient and outpatient chemical-dependency treatment centers (including both privately and publicly funded programs). The investigators obtained the probands’ permission to contact first-degree relatives and invited those family members to participate in the study. In addition, the study included a community-based comparison group that was recruited from a variety of sources, such as dental clinics, health maintenance organizations, and a driver’s license registry. Control families with alcohol-dependent family members were not excluded from the study. This comparison group provided baseline measurements of alcohol dependence and related characteristics in the general population.

The recruitment and assessment of study participants occurred in two stages. In stage I, the researchers recruited pro-bands and family members as previously described and interviewed all participants to determine the presence of alcohol-use disorders, smoking, other drug dependence, and psychiatric disorders. The investigators used a diagnostic interview tool called the Semi-Structured Assessment for the Genetics of Alcoholism (SSAGA), which had been developed specifically for this study (Bucholz et al. 1994; Hesselbrock 1999). Based on the interview, alcohol dependence was defined as meeting lifetime criteria for alcohol dependence as specified in the APA’s (1987) DSM–III–R and the Feighner criteria for definite alcoholism (Feighner et al. 1972). (For a listing of the APA and Feighner criteria, see the textboxes below and on p. 236, respectively.)

Criteria for Alcohol Dependence as Defined by DSM–III–R*

A. At least three of the following:

- Alcohol often taken in larger amounts or over a longer period than the person intended
- Persistent desire or one or more unsuccessful efforts to cut down or control alcohol use
- A great deal of time spent in activities necessary to obtain alcohol, take alcohol, or recover from its effects
- Frequent intoxication or withdrawal symptoms when expected to fulfill major role obligations at work, school, or home (e.g., does not go to work because hung over, goes to school “high,” intoxicated while taking care of his or her children), or when alcohol use is physically hazardous (e.g., drives when intoxicated)
- Important social, occupational, or recreational activities are given up or reduced because of alcohol use
- Continued alcohol use despite knowledge of having a persistent or recurrent physical or psychological problem that is caused or exacerbated by alcohol (e.g., having an ulcer made worse by drinking)
- Marked tolerance: need for markedly increased amounts of alcohol (i.e., at least a 50-percent increase) in order to achieve intoxication or desired effect, or markedly diminished effect with continued use of the same amount
- Characteristic withdrawal symptoms
- Alcohol often taken to relieve or avoid withdrawal symptoms.

B. Some symptoms of the disturbance have persisted for at least 1 month or have occurred repeatedly over a longer period of time.

*DSM–III–R = *Diagnostic Statistical Manual, Third Edition, Revised.*SOURCE: American Psychiatric Association 1987.

**Feighner’s Definition of Alcoholism**

A definite diagnosis is made when symptoms occur in at least three of the four following groups:

**A. Group One**

- Any manifestation of alcohol withdrawal, such as tremulousness, convulsions, hallucinations, or delirium
- History of medical complications, such as cirrhosis, gastritis, pancreatitis, myopathy, polyneuropathy, or Wernicke-Korsakoff’s syndrome
- Alcoholic blackouts (i.e., amnesic episodes during heavy drinking not accounted for by head trauma)
- Alcoholic binges or benders (i.e., 48 hours or more of drinking associated with default of usual obligations; must have occurred more than once to be scored as positive)

**B. Group Two**

- Patient has not been able to stop drinking when he wanted to do so
- Patient has tried to control drinking by allowing himself to drink only under certain circumstances, such as only after 5:00 p.m., only on weekends, or only with other people
- Drinking before breakfast
- Drinking non-beverage forms of alcohol (e.g., hair oil, mouthwash, Sterno, and so forth)

**C. Group Three**

- Arrests for drinking
- Traffic difficulties associated with drinking
- Trouble at work because of drinking
- Fighting associated with drinking

**D. Group Four**

- Patient thinks he drinks too much
- Family objects to his drinking
- Loss of friends because of drinking
- Other people object to his drinking
- Feels guilty about his drinking

In the initial assessment, the researchers did not evaluate the participants for nicotine dependence, but instead used habitual smoking as a proxy, which was defined as smoking 1 pack (i.e., 20 cigarettes) per day for at least 6 months. In a subset of study participants, however, the investigators conducted a full evaluation of nicotine dependence and found that 71 percent of habitual smokers also were nicotine dependent. The investigators used the information from the stage I sample to examine the risk of developing alcohol dependence or habitual smoking in families with alcohol-dependent members, as described in the following section.

To conduct more detailed analyses, the researchers subsequently selected a subset of families with at least three alcohol-dependent members as the stage II sample. For these families, the investigators also recruited more distant biological family members if a member from that branch of the family was alcohol dependent. For all stage II participants, researchers completed a more detailed evaluation and collected blood samples to obtain DNA for genetic studies. Thus, this stage II group of families that were severely affected with alcohol dependence was used for the genetic studies of alcohol dependence and habitual smoking discussed later in this article.

## Transmission of Alcohol Dependence and Habitual Smoking in Families

The familial transmission of alcohol dependence and habitual smoking was examined in stage I families, specifically in the alcohol-dependent probands and their full siblings. The researchers did not include parents or children of the probands in this analysis in order to minimize external influences resulting from changes in drinking and smoking patterns across generations (e.g., changes in general attitudes toward drinking and smoking).

The study found that siblings of alcohol-dependent probands reported substantially higher rates for both alcohol dependence and habitual smoking than did the siblings of control probands (Bierut et al. 1998). For example, almost one-half of the brothers and one-fourth of the sisters of alcohol-dependent probands had a lifetime diagnosis of alcohol dependence, rates that significantly exceeded those found in the general population (i.e., among the control sample) (see table 1). Similarly, habitual smoking was more common in the siblings of alcohol-dependent probands compared with the siblings of control probands. Thus, approximately 42 percent of brothers and 30 percent of sisters of alcohol-dependent probands reported habitual smoking, which again was significantly higher than among the siblings of control probands.

To examine whether the increased rate of habitual smoking among the siblings of alcoholic probands was mediated through an increased rate of alcohol dependence among those siblings themselves, the investigators examined the smoking rates of alcohol-dependent and non-alcohol-dependent siblings of alcoholics. If the increased rate of habitual smoking seen in siblings of alcoholics was mediated only through an increased rate of alcohol dependence, one would expect the rate of habitual smoking in the non-alcohol-dependent siblings to be the same as in the comparison sample. The study found, however, that compared with the community-based sample, an increased risk of habitual smoking existed even among non-alcohol-dependent siblings (see table 2), suggesting that factors in addition to an increased rate of alcohol dependence accounted for the siblings’ risk of being habitual smokers. Indeed, part of this increased risk depended on the alcoholic proband also being a habitual smoker—that is, siblings of smoking alcoholic probands were at higher risks of smoking themselves than were siblings of nonsmoking alcoholic probands, indicating in part a specific familial transmission of habitual smoking. Siblings share many characteristics that may be risk factors for the development of alcohol dependence and smoking. The COGA investigators therefore analyzed the data to evaluate the effects of multiple potentially confounding characteristics on the risk of developing habitual smoking or alcohol dependence. According to these analyses, characteristics that strongly predicted an increased risk of alcohol dependence included the sibling’s gender, birth cohort, and history of other drug dependence, as follows (also see table 3):

- Sisters of alcoholic probands were significantly less likely to be alcohol dependent than were brothers. Furthermore, sisters were less likely to be habitual smokers compared with brothers; however, this difference was not as pronounced as with alcohol dependence.
- Birth cohort significantly influenced the rates of alcohol dependence and habitual smoking among the siblings of alcoholic probands. For example, siblings born between 1950 and 1959 were approximately 63 percent as likely, and siblings born in 1960 or later were approximately 50 percent as likely, to become habitual smokers than were siblings born before 1950. Conversely, alcohol dependence became more common in more recent generations. Thus, siblings born between 1950 and 1959 were 1.5 times more likely, and siblings born in 1960 or later were two times more likely, to be diagnosed with alcohol dependence than were siblings born before 1950. These findings are consistent with the strong trends in smoking and alcohol dependence in the general population, for which the rate of smoking has been decreasing and the rate of alcohol dependence has been increasing in younger generations.
- A diagnosis of one type of drug dependence increased a sibling’s risk of also being diagnosed with dependence on another drug. For example, alcohol-dependent siblings had more than twice the lifetime risk of being habitual smokers^3^ compared with non-alcohol-dependent siblings. Similarly, siblings who were habitual smokers were almost twice as likely to be alcohol dependent than were nonsmokers.

Even after controlling for the influences of individual factors, such as gender, birth cohort, and other drug dependence, however, evidence indicated a familial transmission of a drug-specific risk. Thus, habitual smoking in the probands specifically increased the risk of habitual smoking in their siblings, and alcohol dependence in the probands specifically increased the same diagnosis in their siblings. These findings are consistent with the presence of both common and drug-specific influences that are transmitted in families and combine to determine a person’s risk for developing alcohol dependence or habitual smoking. These influences likely include genetic factors, which are discussed in the following sections.

## Candidate Gene Studies of Alcohol Dependence and Nicotine Dependence

Researchers have used several approaches in trying to identify the genes that influence the susceptibility to a disease, such as alcoholism. One such approach is to study whether an association exists between certain genes that are suspected (based on results of other studies) to play a role in disease development (i.e., candidate genes) and actual disease development. Such association studies evaluate whether a particular form or variant of a candidate gene is more frequently observed in people with the disease than in people without the disease (i.e., control subjects). This association implies that the candidate gene is directly involved in the development of the disease and is “linked” in genetic studies.

However, the association between a candidate gene and the disease may be spurious—that is, the gene actually is not genetically linked to the disease. For example, if the control population is not properly matched (e.g., with respect to ethnicity) to the population from which the affected people are drawn, spurious linkage may occur due to a phenomenon called population stratification. This means that for some genes the frequency with which different gene variants occur in a population may differ between ethnic groups. Consequently, if the samples of people with and without the disease do not have the same ethnic composition, it may appear as if certain gene variants are associated with the disease when, in fact, the frequency of those variants is determined by ethnicity and is unrelated to the disease under investigation. In the COGA study, however, researchers can study candidate genes for both alcohol dependence and habitual smoking without running the risk of spurious findings resulting from population stratification by using family-based tests, such as the Transmission Disequilibrium Test (TDT).^4^ This approach eliminates the need for a separate, matched unaffected control population. As a result, the investigators can directly test for both genetic linkage and association between a gene of interest and the presence of alcohol dependence and habitual smoking.

One candidate gene that has been studied by the COGA investigators is the dopamine D_2_ receptor gene (DRD_2_). Dopamine is a brain chemical (i.e., neurotransmitter) that is used by nerve cells in the brain’s “reward center” and other brain regions to transmit nerve signals among nerve cells. During this process, dopamine is released from the signal-emitting nerve cell and attaches to a protein “docking” molecule (i.e., receptor) located on the outer surface of the signal-receiving nerve cells. This interaction triggers electrical and chemical changes in the signal-receiving cell, resulting in the generation of a new nerve signal in that cell. Several types of dopamine receptors exist, including the DRD_2_ receptor. Furthermore, several variants (i.e., polymorphisms) of the DRD_2_ receptor exist.

Several findings have suggested DRD_2_ as a potential candidate gene that might contribute to the genetic susceptibility to various types of dependence. For example, the dopamine system in general is involved in dependence on a variety of drugs (Smith et al. 1992). Moreover, numerous association studies have specifically implicated the DRD_2_ gene in the development of alcohol dependence (see Edenberg et al. 1998) as well as in smoking (Spitz et al. 1998). These findings, particularly regarding the association of DRD_2_ with alcohol dependence, are controversial, however. Many other studies found no associations between DRD_2_ polymorphisms and alcohol dependence, and linkage studies of DRD_2_ and alcohol dependence also yielded negative results (see Edenberg et al. 1998).

To address this controversy, COGA investigators studied two polymorphisms in separate regions of the DRD_2_ gene in the stage II COGA families using family based association study methods (Edenberg et al. 1998). Neither of those polymorphisms showed any evidence of linkage with alcohol dependence, even when the analysis was limited to more severe forms of alcoholism, where associations should be detectable more easily. Similarly, no significant linkage existed between the DRD_2_ gene and habitual smoking (Bierut et al. 2000).

As mentioned earlier, alcohol dependence and smoking co-occur more often than by chance alone; moreover, twin studies have supported the role of common genetic factors in the development of both disorders. Accordingly, the COGA investigators also examined the association between the DRD_2_ gene and co-occurring alcohol dependence and habitual smoking. Again, those analyses found no evidence that the DRD_2_ gene is linked to the combined phenotype (Bierut et al. 2000). In summary, although the dopamine neuro-transmitter system clearly plays a role in alcohol and other drug use and related disorders, the COGA study detected no association between a genetic variation in the DRD_2_ gene and the development of alcohol dependence and/or habitual smoking. The investigators are currently using the COGA sample to examine additional candidate genes for alcohol dependence and habitual smoking.

## Genomic Screening by Linkage Analysis

In addition to pursuing the candidate gene approach when searching for susceptibility genes for alcohol dependence, researchers have undertaken a more comprehensive search of the entire genome. In the cell, the DNA is packaged into segments, or chromosomes. Each human cell contains 23 pairs of chromosomes,^5^ one set inherited from the mother and one from the father. To screen the entire genome for DNA sequences related to a disease, scientists first identify numerous DNA sequences (which may or may not contain genes) whose locations on the chromosomes are known and which can serve as genetic markers. The goal is to identify enough markers to cover several regions on each chromosome. Subsequently, the researchers test how commonly these markers are shared by siblings who are both affected with alcohol dependence or habitual smoking. If a certain marker is shared more often by affected siblings than can be expected by chance alone, the marker is said to be “linked” with the disease.

The COGA investigators divided their samples into two groups—an initial study sample and a larger replication sample—and conducted independent genomic screens with both groups. The initial sample entailed a complete genome survey of 987 members of 105 multi-generational families that were severely affected with alcohol dependence. The investigators then analyzed 292 genetic markers located across the genome in 382 affected sibling pairs. This analysis found evidence that specific markers located on chromosomes 1, 2, and 7 were linked with alcohol dependence (Reich et al. 1998), thereby suggesting multiple DNA regions that then could be searched in more detail to identify specific candidate genes for the development of alcohol dependence.

To confirm these findings, the investigators conducted a genome survey in the replication sample, which included 1,219 people from 152 families, using 351 genetic markers (Foroud et al. 2000). This analysis found the following results:

- Consistent with the data from the initial sample, there was evidence of linkage (i.e., increased sharing in sibling pairs) of several markers on chromosome 1 with alcohol dependence in the replication sample. This linkage can be expressed in the form of a so-called logarithm-of-the-odds-of-linkage (LOD) score. Higher values of that score indicate stronger evidence for genetic linkage. Thus, the maximum (i.e., peak) LOD score for these markers was 2.5 in the initial sample and 1.6 in the replication sample, suggestive of genetic linkage.
- In contrast with the initial sample, which had shown increased sharing in affected siblings of certain markers on chromosome 2 (peak LOD score 3.0), no evidence of linkage of those markers with alcohol dependence existed in the replication sample (peak LOD score 0.0).
- In the initial sample, there was evidence of genetic linkage between a large area of chromosome 7 and alcohol dependence (peak LOD score 1.6). A smaller part of chromosome 7 also appeared to be linked with alcohol dependence in the replication sample (peak LOD score 1.3), suggestive of genetic linkage.
- Certain markers on chromosome 3, which showed no evidence of linkage in the initial sample (peak LOD score 0.1), showed strong linkage with alcohol dependence in the replication sample (peak LOD score 3.4).

In summary, the COGA study provided evidence that certain regions of chromosomes 1 and 7 were linked with alcohol dependence in both the initial and replicate samples. Other DNA regions, however, may have contained susceptibility genes that were present in only one of the samples (i.e., a region on chromosome 2 in the initial sample and a region on chromosome 3 in the replication sample). It is still unclear why these differences in susceptibility genes exist, particularly because there are no prominent differences between the initial sample and the replication sample. One can speculate that many genes of modest effect influence the development of alcohol dependence. Because such genes will be difficult to detect consistently, it is possible that one gene may be identified in the initial sample and a different gene may be identified in the replication sample.

The COGA genome screen data were also used in the Genetic Analysis Workshop (GAW),^6^ conducted in Aracachon, France, in September 1998. During that workshop, the researchers compared the genome data with the COGA participants’ lifetime histories of smoking and alcohol dependence. This analysis identified several DNA regions that appeared to show shared linkage with both disorders (Bergen et al. 1999). The genetic study of co-occurring habitual smoking and alcohol dependence is ongoing.

In summary, the COGA investigators have completed a comprehensive search for the genetic linkage of certain DNA regions with alcohol dependence and habitual smoking. These analyses support the hypothesis that some common genetic factors are involved in the susceptibility for developing both disorders. Additionally, these data provide evidence that specific genetic factors may be involved in the development of alcohol dependence and habitual smoking.

## Future Directions

Numerous developments, such as the increasing identification of human genes and their functions, the sequencing of the entire human genome, and the development of new analytic techniques to detect linkage, have resulted in a surge in genetic studies. These advances provide the tools that allow researchers to study the genetic basis of common, complex disorders, such as smoking and alcohol dependence. In addition to these ongoing genetic analyses, researchers are exploring other characteristics thought to be related to the susceptibility for developing alcohol-related problems, including personality traits and various measures of the brain’s electrical activity (e.g., electroencephalograms and event-related potentials). An integration of these results from multiple correlated domains, along with further genetic findings, will improve researchers’ understanding of the mechanisms and risk factors underlying the development of alcohol dependence and habitual smoking.

## Figures and Tables

**Table 1:** Demographic Characteristics and Lifetime Prevalences of Alcohol Dependence and Habitual Smoking in Probands and Siblings

	COGA Probands (*n* = 1,212)	COGA Siblings (*n* = 2,755)	Control Probands (*n* = 217)	Control Siblings (*n* = 254)
**Gender**				
Men (*n*)	916*	1,197	111	111
Women (*n*)	296*	1,558	106	143
**Age**				
Mean (years)	37.72*	36.35**	34.57	26.15
Range (years)	17–77	18–76	18–68	17–48
**Alcohol Dependence**				
Men (%)	100.0*	49.7**	16.2	19.8
Women (%)	100.0*	23.8**	03.8	06.0
**Habitual Smoking**				
Men (%)	63.0*	41.6**	18.0	13.5
Women (%)	55.0*	30.4**	12.3	09.8

* Statistically significant difference (i.e., *p* < 0.001) versus control probands.

** Statistically significant difference (i.e., *p* < 0.001) versus control siblings.

COGA = Collaborative Study on the Genetics of Alcoholism.

Probands = alcohol-dependent patients and control subjects initially recruited for the COGA study.

SOURCE: Adapted from Bierut et al. 1998.

**Table 2:** Lifetime Rates of Habitual Smoking in Alcohol-Dependent and Non-Alcohol-Dependent Siblings of COGA Probands

	Alcohol-Dependent Siblings of COGA Proband		Non-Alcohol-Dependent Siblings of COGA Proband	

Habitual Smoking	Male (%)	Female (%)	Male (%)	Female (%)
Yes	61.3	60.7	35.7	29.9
No	40.9	37.3	20.3	14.6

COGA = Collaborative Study on the Genetics of Alcoholism.

Probands = alcohol-dependent patients and control subjects initially recruited for the COGA study.

SOURCE: Adapted from Bierut et al. 1998.

**Table 3:** Factors Affecting the Risk of Developing Alcohol Dependence and Habitual Smoking in Siblings of Alcohol-Dependent COGA Probands

	Risk Ratio (95% Confidence Interval)	

	Alcohol Dependence	Habitual Smoking
**Sibling Characteristics**		

Birth Cohort		
Born before 1950	1.00	1.00
Born 1950–1959	1.53 (1.23–1.90)	0.63 (0.53–0.77)
Born 1960 or later	2.07 (1.65–2.60)	0.50 (0.41–0.61)
Gender		
Male	1.00	1.00
Female	0.48 (0.42–0.55)	0.86 (0.75–0.98)
Alcohol Dependence	NA	2.06 (1.78–2.39)
Habitual Smoking	1.92 (1.68–2.20)	NA
**Proband Characteristics**		

Gender		
Male	1.00	1.00
Female	1.09 (0.94–1.26)	1.09 (0.91–1.30)
Alcohol Dependence	2.60 (1.60–4.01)	1.66 (0.99–2.79)
Habitual Smoking	0.98 (0.85–1.12)	1.77 (1.48–2.12)

COGA = Collaborative Study on the Genetics of Alcoholism.

Probands = alcohol-dependent patients and control subjects initially recruited for the COGA study.

SOURCE: Adapted in part from Bierut et al. 1998.

## Footnotes

1 According to epidemiologic analyses, approximately one-third of all tobacco users are nicotine dependent (see the article in this issue by Anthony and Echeagaray-Wagner, pp. 201–208).

2 A genetic correlation can range from 0 (i.e., no overlap between the genetic factors determining both disorders) to 1.0 (i.e., complete overlap of the genetic factors).

3 Because the COGA researchers did not have sufficient information to determine nicotine dependence in the probands and siblings, they used the characterization as a habitual smoker as a proxy for dependence, particularly because, as stated earlier, approximately 70 percent of habitual smokers are indeed nicotine dependent.

4 In this test, the two different variants (i.e., alleles) of a gene that a person inherits from each parent are examined to determine which allele has been transmitted to an affected offspring. The nontransmitted allele serves as a control for the transmitted allele.

5 Using certain techniques, scientists can visualize the chromosomes under a microscope and order and number them according to their size, with chromosome 1 being the largest chromosome.

6 These workshops are held every 2 years to foster the development of new genetic methods and to apply these methods to both real and simulated data.
